# BMP‐Smad Signaling Regulates Postnatal Crown Dentinogenesis in Mouse Molar

**DOI:** 10.1002/jbm4.10249

**Published:** 2019-11-14

**Authors:** Maiko Omi, Anshul K Kulkarni, Anagha Raichur, Mason Fox, Amber Uptergrove, Honghao Zhang, Yuji Mishina

**Affiliations:** ^1^ Department of Biologic and Materials Sciences and Prosthodontics University of Michigan School of Dentistry Ann Arbor MI USA

**Keywords:** BMP, DENTAL MESENCHYME, DENTIN, DENTINOGENESIS, ODONTOBLAST

## Abstract

Dentinogenesis, a formation of dentin by odontoblasts, is an essential process during tooth development. Bone morphogenetic proteins (BMPs) are one of the most crucial growth factors that contribute to dentin formation. However, it is still unclear how BMP signaling pathways regulate postnatal crown and root dentinogenesis. BMPs transduce signals through canonical Smad and non‐Smad signaling pathways including p38 and ERK signaling pathways. To investigate the roles of Smad and non‐Smad signaling pathways in dentinogenesis, we conditionally deleted *Bmpr1a*, which encodes the type 1A receptor for BMPs, to remove both Smad and non‐Smad pathways in *Osterix*‐expressing cells. We also expressed a constitutively activated form of *Bmpr1a* (*caBmpr1a*) to increase Smad1/5/9 signaling activity without altered non‐Smad activity in odontoblasts. To understand the function of BMP signaling during postnatal dentin formation, Cre activity was induced at the day of birth. Our results showed that loss of BmpR1A in odontoblasts resulted in impaired dentin formation and short molar roots at postnatal day 21. *Bmpr1a* cKO mice displayed a reduction of dentin matrix production compared to controls associated with increased cell proliferation and reduced *Osx* and *Dspp* expression. In contrast, *caBmpr1a* mutant mice that show increased Smad1/5/9 signaling activity resulted in no overt tooth phenotype. To further dissect the functions of each signaling activity, we generated *Bmpr1a* cKO mice also expressing *caBmpr1a* to restore only Smad1/5/9 signaling activity. Restoring Smad activity in the compound mutant mice rescued impaired crown dentin formation in the *Bmpr1a* cKO mice; however, impaired root dentin formation and short roots were not changed. These results suggest that BMP‐Smad signaling in odontoblasts is responsible for crown dentin formation, while non‐Smad signaling may play a major role in root dentin formation and elongation. © 2019 The Authors. *JBMR Plus* published by Wiley Periodicals, Inc. on behalf of American Society for Bone and Mineral Research. © 2019 The Authors. *JBMR Plus* published by Wiley Periodicals, Inc. on behalf of American Society for Bone and Mineral Research.

## Introduction

Dentinogenesis begins at the late bell stage of tooth development. Odontoblasts, the cells of mesenchymal origin, located underneath the enamel, play an essential role in dentin formation. Dentin is classified as primary, secondary, and tertiary dentin by their location, timing of development, and histological characteristics.^1^ Primary dentin is formed during early tooth development, whereas secondary dentin is produced after the tooth becomes functional. Tertiary dentin is secreted in response to external stimulation such as cavities and wear. Odontoblasts actively secrete dentin matrix during primary dentinogenesis, but they become less active during secondary dentinogenesis. The tertiary dentin produced by pre‐existing or newly differentiated odontoblasts is deposited rapidly with irregular tubular patterns and some cellular inclusions called osteodentin. Unlike mouse incisors that show continuous growth, mouse molars are similar to human teeth as they do not show continuous growth and are composed of two parts: crown and root. Recent studies provide evidence that formation of crown and root undergoes distinct cellular and molecular regulatory mechanisms.^2^, ^3^ However, it is not well understood whether odontoblasts residing in either crown or root have unique site‐specific functions in dentinogenesis.

Bone morphogenetic proteins (BMPs), members of the transforming growth factor‐β (TGF‐β) superfamily, are one of the crucial factors for tooth morphogenesis. Previous studies reported that BMPs secreted from the dental mesenchyme are critical for odontoblast and ameloblast differentiation during early tooth morphogenesis.^4^, ^5^ We and others demonstrated that disruption of *Bmp2* or *Bmp4* in odontoblasts results in impaired crown and root dentin formation.^6^, ^7^ These findings suggest that BMP signaling also plays an important role in postnatal tooth development. BMP ligands bind to heterotetrameric receptor complexes consisting of two type 1 receptors and two type 2 receptors. These complexes activate type 1 receptors and transduce intracellular signaling through Smad1/5/9 proteins. BMP signaling is also mediated through non‐Smad pathways (ie, Smad independent pathways or noncanonical BMP signaling pathways) including p38 and ERK signaling pathways.^8^, ^9^ It has been demonstrated that Smad4 mediated by TGF‐β/BMP signaling plays an important role in tooth development.^10^, ^11^, ^12^ However, how BMP‐Smad and BMP‐non‐Smad signaling coordinately regulate postnatal crown and root dentin formation has not been elucidated.

In this study, we investigated how BMP signaling regulates postnatal dentinogenesis by altering BMP‐Smad and BMP‐non‐Smad signaling activity in odontoblasts. Our results suggest that BMP‐Smad signaling in odontoblasts regulates crown dentinogenesis, whereas non‐Smad signaling may play a major role in root dentinogenesis during postnatal tooth development.

## Materials and Methods

### Animals

To generate *Bmpr1a* cKO mice, mice heterozygous null for *Bmpr1a* carrying the Tet‐off *Osterix*‐*Cre* (*Bmpr1a* ^*+/−*^ *;Osx‐Cre*)^13^, ^14^ were bred with mice homozygous for the conditional allele for *Bmpr1a* (*Bmpr1a* ^*fx/fx*^ *;R26R/R26R*). Mice genotyped *Bmpr1a* ^*fx/+*^ *;Osx‐Cre;R26R/+* and *Bmpr1a* ^*fx/−*^ *;Osx‐Cre;R26R/+* were designated as control and *Bmpr1a* cKO, respectively. Mice conditionally express a constitutively active form of *Bmpr1a* (*caBmpr1a*), which has a mutation in Q227D resulting in ligand‐independent activation of BMP‐Smad signaling after Cre recombination^15^, ^16^ were crossed with *Osx*‐*Cre* mice to generate *caBmpr1a* ^*+/−*^;*Osx‐Cre* mice. For rescue experiments, *caBmpr1a* ^*+/−*^ *;Bmpr1a* ^*fx/fx*^ mice were bred with *Bmpr1a* ^*+/−*^ *;Osx‐Cre* mice to obtain compound mutant mice (*caBmpr1a* ^*+/−*^ *;Bmpr1a* ^*fx/−*^ *;Osx‐Cre*). To suppress Cre activity at the embryonic stage, pregnant mothers were fed a diet containing 625 mg/kg doxycycline (Harlan, Indianapolis, IN, USA) to deliver a daily dose of 2 to 3 mg/mouse of doxycycline. Cre activity was activated at the day of birth by switching from doxycycline chow to regular rodent diet of nursing mothers. To monitor the activity of Cre recombinase, *Osx‐Cre* mice were crossed with *R26* ^*mTmG*^ mice. All animal experiments in this study were approved by the Institutional Animal Care and Use Committee (IACUC) at the University of Michigan and were conducted accordance with ARRIVE guidelines.

### Micro‐computed tomography (micro‐CT)

For micro‐CT analysis, right mandibulae were placed in a 19‐mm‐diameter specimen holder and scanned over the entire length of the mandible using a micro‐CT system (μCT100 Scanco Medical, Bassersdorf, Switzerland). Scan settings were: voxel size 12 μm, 70 kVp, 114 μA, 0.5 mm AL filter, and integration time 500 ms. Mesial root height and volume measurements of the enamel, crown dentin, and root were taken from the first molar using ITK‐SNAP^17^ with at least three independent litters at postnatal day 21 (P21) including both male and female (*n* = 6 per group).

### Histology and histomorphometry

Left mandibulae were fixed in 4% paraformaldehyde, decalcified with 10% EDTA, and embedded in paraffin. A series of coronal and sagittal sections was made at 5 μm and stained for hematoxylin and eosin (H&E). Histomorphometric analysis of the mesial root of the first molars was made in a blinded, nonbiased manner by using ImageJ (NIH) (*n* = 6 per group). The cervical regions of crown and root thickness were determined from the cemento‐enamel junction that extended 100 μm toward the coronal and apical side, respectively. To measure mineral apposition rates, mice were injected with calcein (10 mg/kg of body weight, i.p.) at P14 and at P19 and euthanized at P21. The distance between the parallel calcein lines was measured by using ImageJ (*n* = 6 per group).

### Immunohistochemistry

Left mandibulae were fixed in 4% paraformaldehyde, decalcified with 10% EDTA, and embedded in paraffin. Deparaffinized sections were placed in 0.05% trypsin for antigen retrieval. After treatment with 3% hydrogen peroxide and blocking solution, the sections were treated with the primary phospho‐Smad1/5/9 (pSmad1/5/9) antibody overnight at 4°C. The sections were treated with HRP‐conjugated goat anti‐rabbit IgG (Abcam, Cambridge, MA, USA) according to the manufacturer's instructions (*n* = 6 per group). For immunofluorescence staining, right mandibulae were decalcified with 10% EDTA and embedded in OCT compound (Thermo Fisher Scientific, Waltham, MA, USA). The sections were incubated overnight at 4°C with antibodies against Ki‐67 and phospho‐Histone H3, and Alexa Fluor 568 (1:200, Invitrogen, Carlsbad, CA, USA) were used for detection (*n* = 6 per group). The details on primary antibodies and dilutions are shown in Supplemental Table S1.

### Quantitative reverse transcription‐polymerase chain reaction (qRT‐PCR)

The first and second molars were dissected out from the mandible and subjected to RNA extraction with TRIzol reagent (Ambion, Austin, TX, USA). cDNA was synthesized from 500 ng of RNA using SuperScript II cDNA Synthesis (Invitrogen) following the manufacturer's instruction. Levels of gene expressions were compared between different genotypes using Applied Biosystems (Carlsbad, CA, USA) ViiA7 platform. Expression levels of each gene were normalized by endogenous GAPDH. The following probes were purchased from Taqman (Applied Biosystems): *Bmpr1a*: Mm00477650_m1; *Acvr1*: Mm01331069_m1; *Bmpr1b*: Mm00432117_m1; and *Gapdh*: Mm99999915_g1. The primers to quantify other gene expression levels using SYBR Green method are shown in Supplemental Table S2. The amplification specificity was confirmed by melting curves (*n* = 6 per group).

### Western blotting

The first and second molars were dissected out from the mandible and frozen in liquid nitrogen. The tissues were crashed in a frozen mortar and pestle and lysed in RIPA buffer (20 mM Tris–HCl, 0.1% SDS, 1% Triton X‐100, 1% sodium deoxycholate). Subsequently, protein lysates were separated by 10% SDS‐PAGE and transferred to PVDF membrane (Millipore, Burlington, MA, USA). Specific bindings were visualized with SuperSignal West Pico Chemiluminescent substrate (Thermo Fisher Scientific). The protein levels of phosphorylated forms of p38 and ERK were normalized to the respective total form, and pSmad1/5/9 were normalized to GAPDH by using ImageJ (*n* = 3 per group). The details on primary antibodies and dilutions are shown in Supplemental Table S1.

### Statistical analysis

Statistical difference between two groups were analyzed using Student's two‐tailed unpaired *t* test, and those among three groups were analyzed using one‐way analysis of variance (ANOVA), followed by a Turkey test. All experiments were performed in three or more biological replicates per group.

## Results

### *Osx*‐expressing odontoblasts differentially contribute to crown and root dentin formation in molars

**Figure 1:**
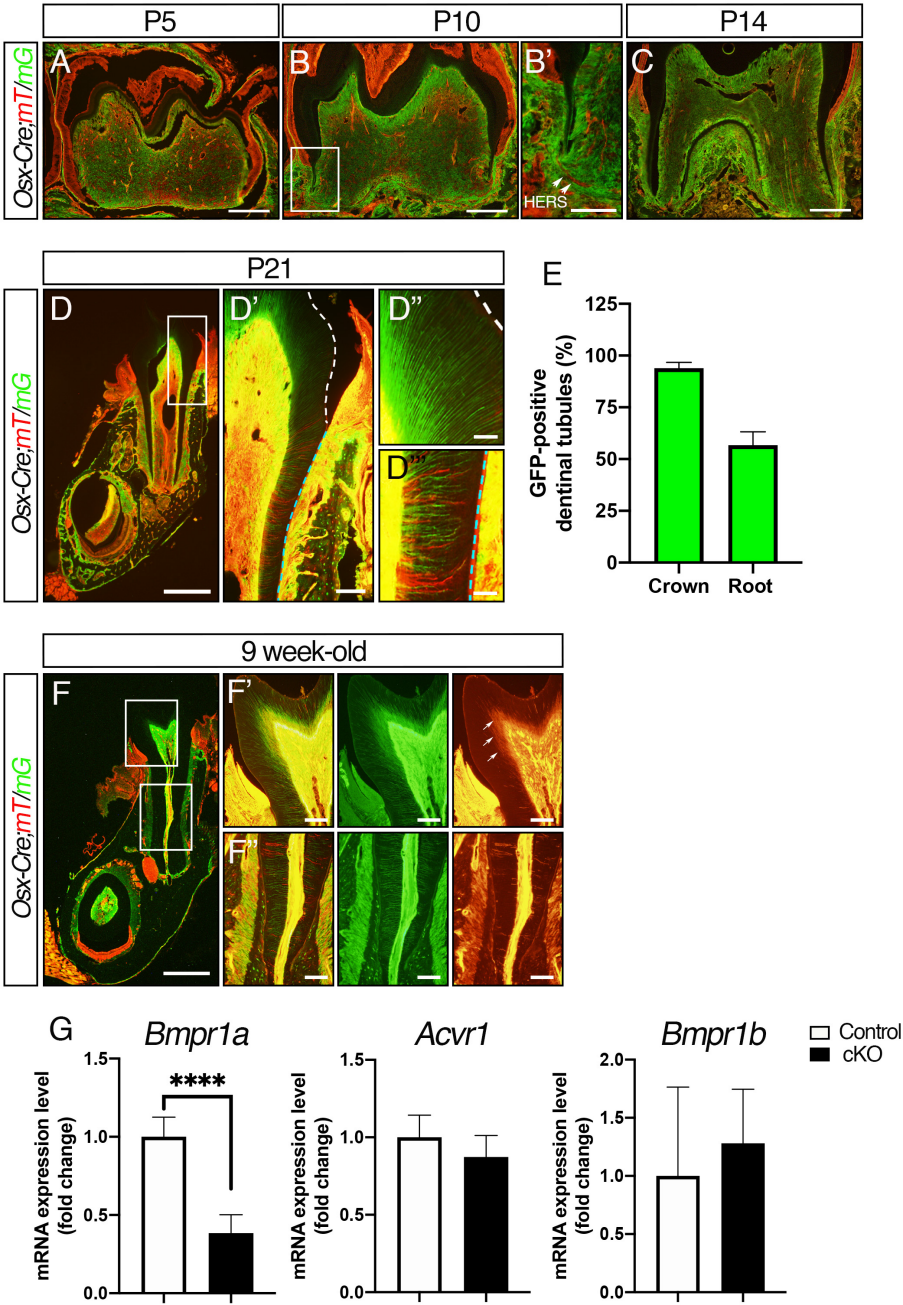
*Osterix*‐expressing odontoblasts differentially contribute to crown and root dentinogenesis in molars. (*A*) The sagittal sections of the first molar in the mandible at postnatal day 5 (P5). Odontoblasts and pulp cells in the crown were GFP (mG)‐positive. (*B*) Tooth elongation initiated at P10. Cells in Hertwig's epithelial root sheath (HERS) were tomato (mT)‐positive (*B’*). (*C*) GFP‐positive cells were located both in crown and root pulp at P14. (*D*) The coronal sections of the mesial root of first molars at P21. GFP‐positive cells were observed in pulp, periodontal ligaments, and alveolar bones. High magnification of cervical (*D’*), crown (*D”*), and root (*D”’*) regions. The white dotted line indicates dentino‐enamel junction (DEJ) and the blue dotted line indicates cemento‐dentin junction (CDJ). (*E*) Quantification of GFP‐positive and GFP‐negative dentinal tubules in the crown and root regions. (*F*) The coronal sections of the mesial root of first molars at 9 weeks. mT‐positive dentinal tubules were observed in secondary dentin in the crown (white arrows). High magnification of crown (*F’*) and root (*F”*) regions. (*G*) Expression levels of *Bmpr1a*, *Acvr1*, and *Bmpr1b* between control and *Bmpr1a* cKO molars were assessed by qRT‐PCR. Data were normalized to the amounts of endogenous *Gapdh*. Values represent the mean ± SD. *n* = 6, ****p < 0.0001. Scale bars = 250 μm (*A*–C), 500 μm (*D–F*), 100 μm (*B’, D’, F’, F”*), 25 μm (*D’, D”*).

*Osx* potentially marks dental mesenchymal progenitors that differentiate into odontoblasts and produce mineralized dentin matrix.^6^, ^18^ We noticed that conditional disruption of *Bmpr1a* using a constitutively active *Osx‐Cre* results in embryonic lethality (data not shown) and thus we decided to use an inducible (Tet‐off) system to activate Cre activity during postnatal development. Since we induced Cre activity at the day of delivery by removing doxycycline, we tested whether Cre recombination occurred efficiently in odontoblasts by using mTmG Cre reporter mice. Before Cre recombination, all cells express a transmembrane form of tdTomato fluorescent protein (mT). After Cre recombination, the mT expression switches to the transmembrane form of GFP (mG). At P5, GFP‐positive odontoblasts were observed in the crown (Fig. 1 *A*). {FIG1} Tooth root formation was initiated at P10, and GFP‐positive cells were observed around roots but cells in Hertwig's epithelial root sheath (HERS) were mT‐positive (Fig. 1 *B, B’*), indicating that *Osx‐Cre* does not mark dental epithelial cells. At P14, GFP‐positive odontoblasts were located both in the crown and root (Fig. 1 *C*). At P21, GFP‐positive cells were observed within pulp, periodontal ligaments, and alveolar bones (Fig. 1 *D*). Most of the dentinal tubules running from crown odontoblasts toward dentino‐enamel junction (DEJ) (Fig. 1 *D’*, *D”*, white dotted line) were GFP‐positive, whereas those from root odontoblasts toward cemento‐dentin junction (CDJ) (Fig. 1 *D’*, *D’”*, blue dotted line) were either GFP‐positive or mT‐positive (Fig. 1 *E*). These results suggest that *Osx*‐expressing odontoblasts contribute to both crown and root dentin formation, but *Osx*‐negative odontoblasts also contribute to root dentin formation. To understand the heterogeneity of odontoblasts during secondary dentinogenesis, we assessed dentin formation at 9 weeks (Fig. 1 *F*). Consistent with the result at P21, most of the dentinal tubules running from crown odontoblasts toward DEJ were GFP‐positive. Interestingly, mT‐positive dentinal tubules running from crown odontoblasts were observed around pulpal horn (Fig. 1 *F’*, white arrows), suggesting that newly differentiated *Osx*‐negative odontoblasts form secondary dentin in the crown. There were some mT‐positive dentinal tubules running from root odontoblasts toward CDJ (Fig. 1 *F”*). These results suggest that homogeneous populations of odontoblasts form primary dentin in the crown, whereas heterogeneous populations of odontoblasts form root dentin as well as secondary dentin.

In addition to monitoring Cre activity by using mTmG Cre reporter, we assessed expression levels of *Bmpr1a* in mandibular molars (Fig. 1 *G*). The molars from the *Bmpr1a* cKO mice resulted in a 62% reduction of *Bmpr1a*. *Osx‐Cre* marks 94% of crown odontoblasts and 57% of root odontoblasts as shown in Fig. 1 *D, E*. Since *Bmpr1a* expression levels in the entire molar reduced by more than 60%, Cre recombination in *Osx*‐expressing cells is highly efficient in the cKO model. Expression levels of the other two type 1 receptors were not different between control and the cKO molars as is the case with osteoblasts that lost *Bmpr1a*.^15^

### Disruption of BmpR1A in odontoblasts results in reduced dentin thickness and short roots

**Figure 2:**
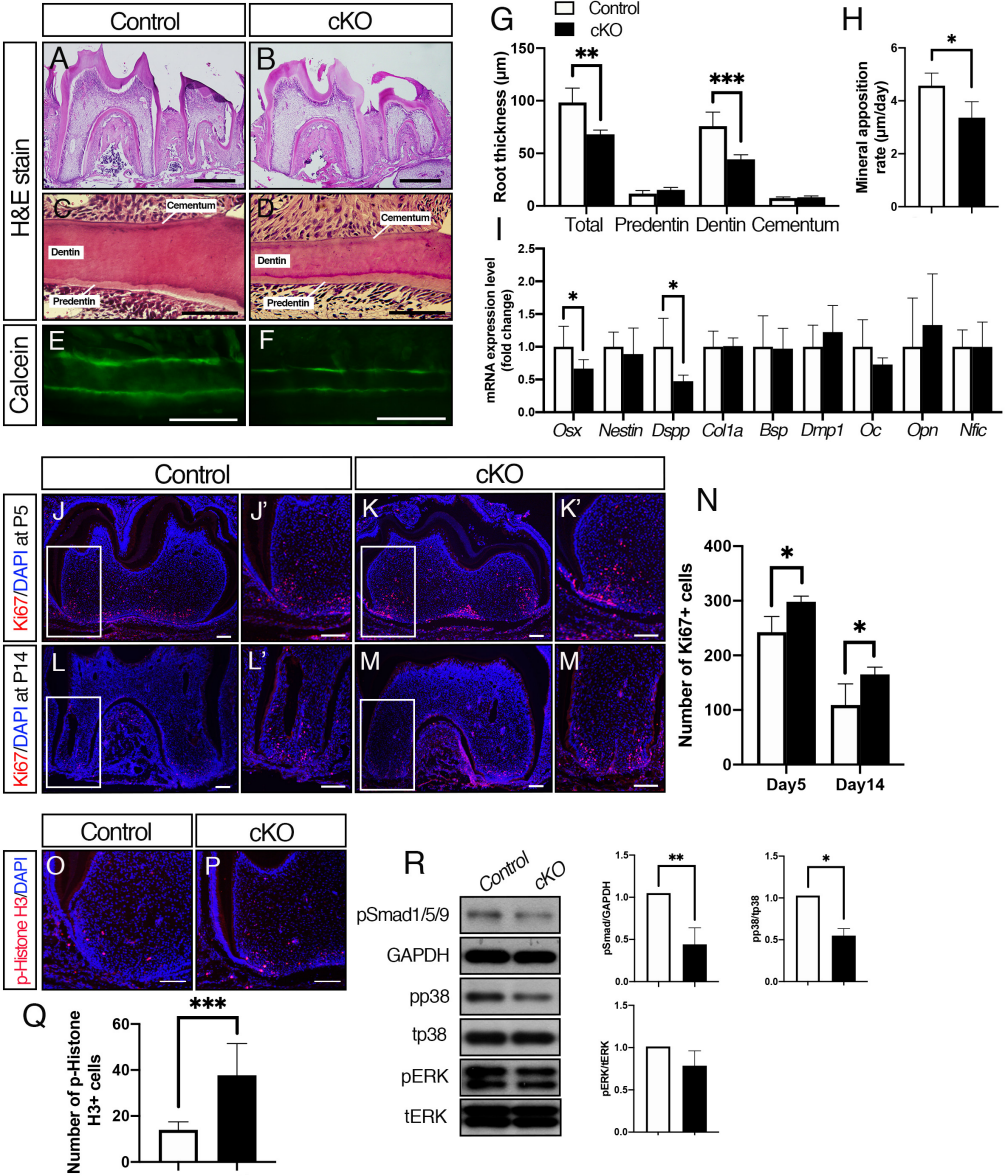
Disruption of BMP signaling in odontoblasts results in reduced dentin formation associated with decreased *Osx* and *Dspp* expression. (*A*, *B*) H&E staining of sagittal sections of the mandibular first molar at P21. (*C*, *D*) Coronal sections of cervical region of the first molar. (*E*, *F*) Calcein labeling of root dentin showed reduced matrix formation in the cKO molars. Calcein was injected at P14 and P19, and tissues were harvested at P21. (*G*) Quantification of root thickness in the cervical region of the first molar. Dentin in the *Bmpr1a* cKO molars was thinner than that in controls. (*H*) Measurements of mineral apposition rate. (*I*) Expression levels of *Osx*, *Nestin*, *Dspp*, *Col1a*, *Bsp*, *Dmp1*, *Osteocalcin* (*Oc*), *Osteopontin* (*Opn*), and *Nfic* in the mandibular molars were measured by qRT‐PCR. The expression levels of *Osx* and *Dspp* were significantly reduced in the cKO molars. (*J–M*) Ki67‐positive cells in the apical region of dental pulp were increased in the cKO molars both at P5 (*J*, *K*) and P14 (*L*, *M*). High magnification of the apical region of dental pulp (*J’*–*M’*). (*N*) Quantification of Ki67‐positive cells in the apical region of dental pulp. (*O*, *P*) Phospho (p)‐histone H3‐positive cells in the apical region of dental pulp were increased in the cKO molars at P5. (*Q*) Quantification of p‐histone H3‐positive cells in the apical region of dental pulp. (*R*) Protein lysates were collected from molars at P21 and subjected to Western blot analysis. The protein levels of phosphorylated forms of p38 and ERK were normalized to the respective total form, and pSmad1/5/9 levels were normalized to GAPDH using ImageJ. *Bmpr1a* cKO molars displayed reduced pSmad1/5/9 and pp38 levels compared with controls. Representative images of protein bands are shown. Values represent the mean ± SD. Differences were assessed by Student's *t* test, *n* = 6 (*A*–*Q*), 3 (*R*), *p < 0.05, **p < 0.01. Scale bars = 500 μm (*A*, *B*), 50 μm (*C–F*), 100 μm (*J–M*, *J’–M’*,*O*, *P*).

To address whether deletion of BMP signaling in odontoblasts affects postnatal tooth formation, we conducted morphological analysis of the mandibular first molar by H&E stain. At P21, the mandibular first molars in the *Bmpr1a* cKO mice displayed short roots and reduced dentin thickness compared with those in controls (Fig. 2 *A*–*D*). {FIG2} The root dentin in the cervical region was thinner, but predentin was thicker in the cKO molars (Fig. 2 *C*, *D*, *G*). No significant difference in cementum thickness was observed between the cKO and control molars. Calcein labeling showed a reduction of mineral apposition rate in the cKO molars (Fig. 2 *E*, *F*,*H*). These findings suggest that BMP signaling in odontoblasts regulates dentin matrix production and mineralization. Next, we evaluated expression levels of odontogenic and osteogenic marker genes in molars by qRT‐PCR. The results showed that expression levels of *Osx* and *Dspp* were significantly reduced in the cKO molars, whereas those of *Nestin*, *Col1a*, *Bsp*, *Dmp1*, *Osteocalcin* (*Oc*), and *Osteopontin* (*Opn*) were not changed in the cKO molars (Fig. 2 *I*). The expression levels of *Nfic*, an important transcription factor that regulates tooth root formation,^2^, ^19^, ^20^ were also unchanged in the cKO molars. Next, we addressed impacts of the loss of BMP signaling on cell proliferation of odontoblast progenitors. The number of Ki67‐positive cells in the pulp was increased in the cKO molars compared with controls at P5 and P14 (Fig. 2 *J*–*M*, *J’*–*M’*, *N*). The number of p‐Histone H3‐positive cells in the pulp was also increased in the cKO molars at P5 (Fig. 2 *O*–*Q*), suggesting that loss of BMP signaling in odontoblast progenitors promotes cell proliferation. The Western blot analysis showed that the cKO molars displayed reduced pSmad1/5/9 and pp38 levels compared with controls (Fig. 2 *R*), suggesting that disruption of *Bmpr1a* in odontoblasts reduces both Smad and non‐Smad signaling, especially p38 signaling activities.

### Increased Smad1/5/9 signaling activity in odontoblasts results in no overt tooth phenotype

**Figure 3:**
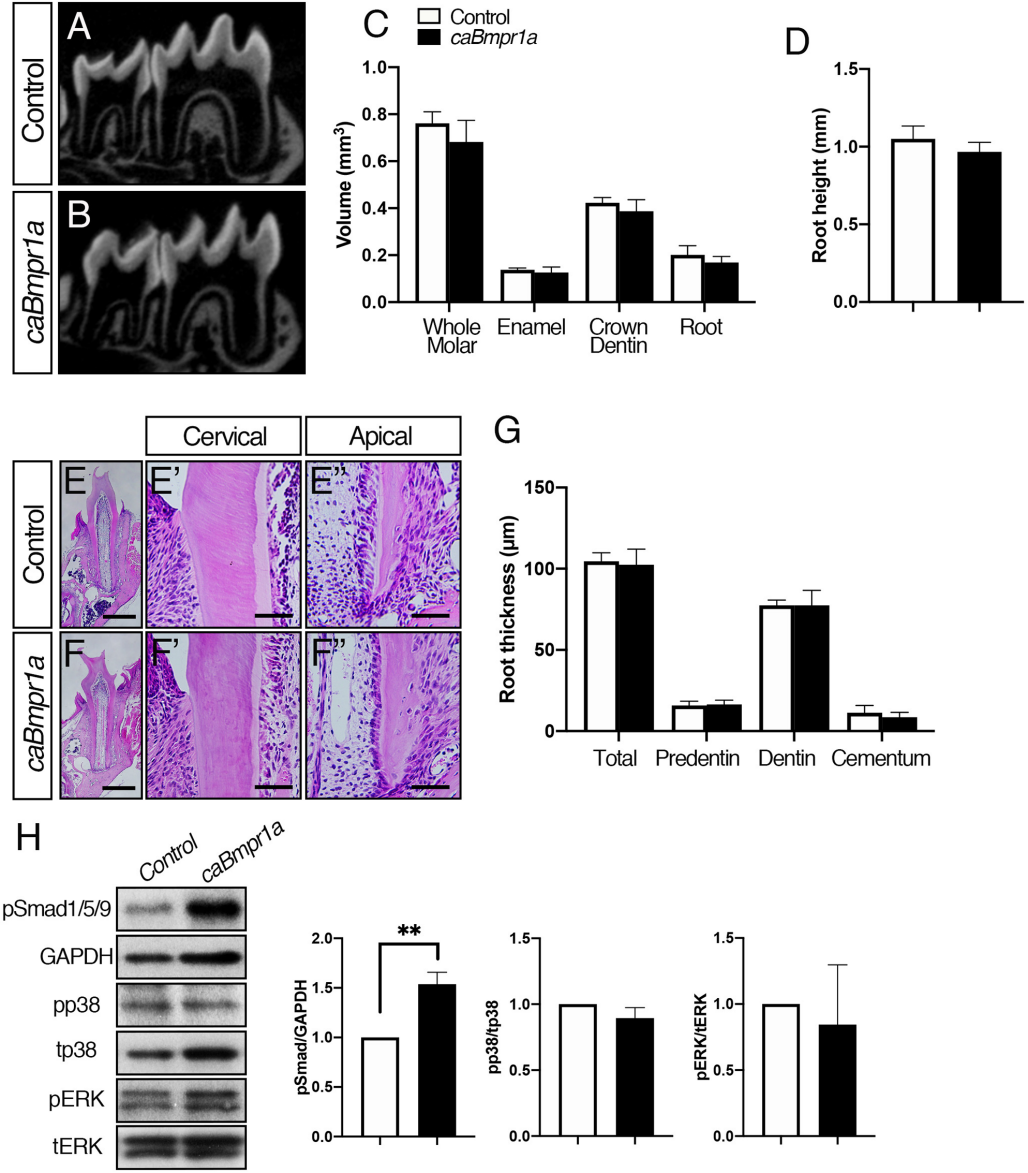
Increased BMP signaling in odontoblasts results in no overt tooth phonotype. (*A*, *B*) Representative sagittal view of micro‐CT images of mandibular molars from control (*A*) and *caBmpr1a* mutant (*B*) mice at P21. (*C*) Volumetric analysis of the mandibular first molars. (*D*) The average heights of mesial roots of the first molars. There was no significant difference between control and *caBmpr1a* mutant molars. (*E*, *F*) Representative coronal images of H&E staining of the first molar at P21. High magnification of the cervical (*E’*, *F’*) and apical (*E”*,*F”*) regions. (*G*) Quantification of root thickness in the cervical region of the first molars. The region was determined from the cemento‐enamel junction that extended 100 μm toward apical direction. (*H*) Protein lysates were collected from molars at P21 and subjected to Western blot analysis. The protein levels of phosphorylated forms of p38 and ERK were normalized to the respective total form, and pSmad1/5/9 levels were normalized to GAPDH using ImageJ. *caBmpr1a* mutant molars displayed increased pSmad1/5/9 levels compared with controls. Representative images of protein bands are shown. Values represent the mean ± SD. Differences were assessed by Student's *t* test, *n* = 6 (*A–G*), 3 (*H*), **p < 0.01. Scale bars = 500 μm (*E*, *F*), 100 μm (*E’*, *F’*, *E”*, *F”*).

To investigate whether enhanced BMP‐Smad signaling influences postnatal dentin formation, we conditionally expressed *caBmpr1a* in *Osx*‐expressing cells. The volumetric analysis demonstrated that there was no significant difference in the crown and root dentin volume, and root heights between *caBmpr1a* mutant and control molars (Fig. 3 *A*–*D*). {FIG3} The H&E stain also showed that there was no significant difference in the dentin thickness between two groups (Fig. 3 *E*–*G*). The Western blot analysis showed that the *caBmpr1a* mutant molars displayed increased pSmad1/5/9 levels without altered non‐Smad signaling pathways such as p38 and ERK (Fig. 3 *H*), suggesting that elevated pSmad1/5/9 activity in odontoblasts does not influence postnatal dentin formation. We previously reported that *caBmpr1a* mutant mice having a *P0‐Cre* transgene display a shorter and broader snout and a deformed skull.^21^, ^22^ We did not observe such craniofacial deformities in the *caBmpr1a;Osx‐Cre* mutant mice.

### Reduced crown dentin volume in *Bmpr1a* cKO molars is partially restored by increased pSmad1/5/9 signaling activity

**Figure 4:**
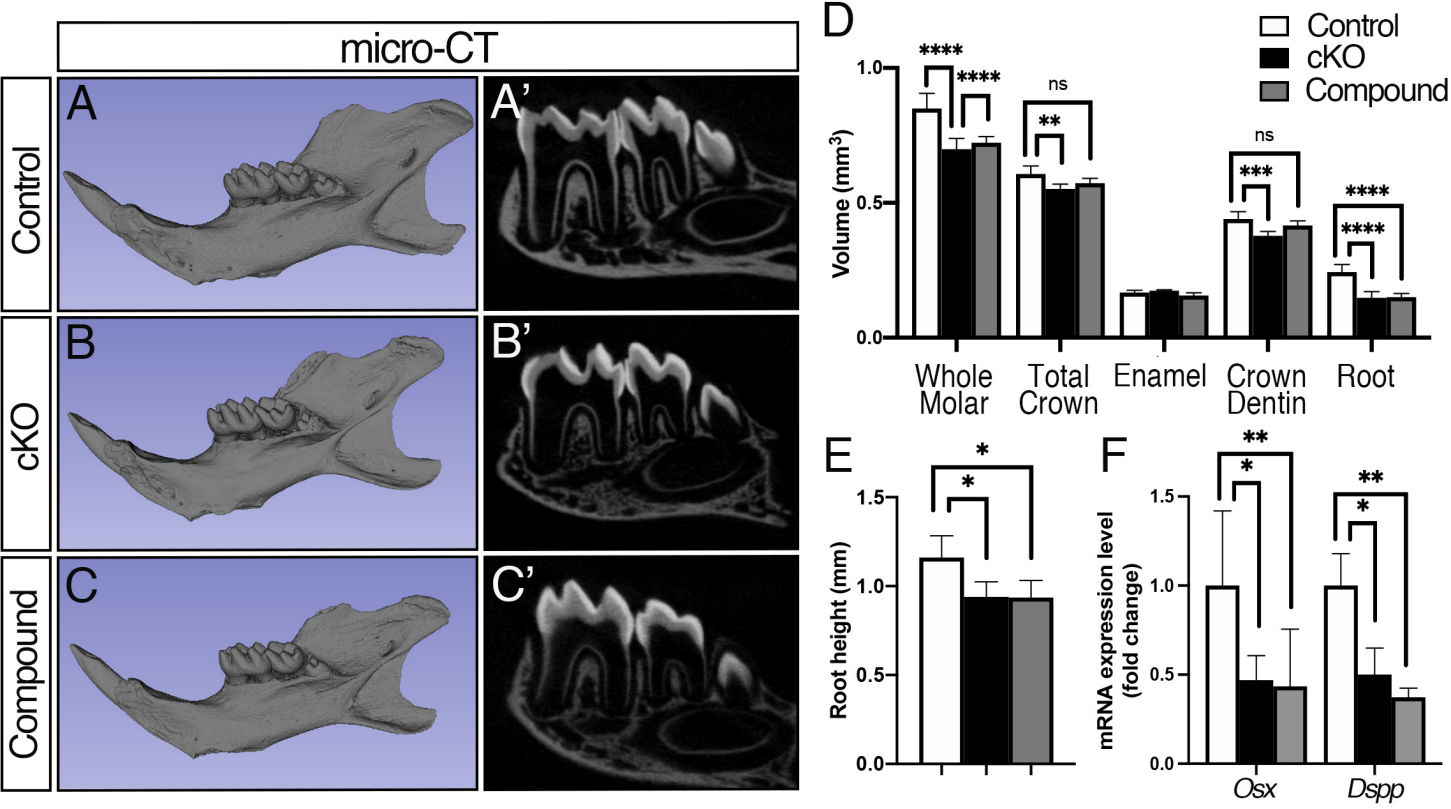
Impaired dentin formation in *Bmpr1a* cKO molars is partially restored in the compound mutant molars. (*A*–*C*) Representative 3D micro‐CT images of mandibles (*A*–*C*) and 2D sagittal images of mandibular molars (*A’*–*C’*). The mandibles from *Bmpr1a* cKO and compound mutant mice resulted in smaller size and less alveolar bones compared with controls. (*D*) Volumetric analysis of the mandibular first molars. Reduced crown dentin volume in *Bmpr1a* cKO molars were partially restored in the compound mutants. (*E*) The average heights of the mesial roots of mandibular first molars. The compound mutant mice displayed a reduction of molar root heights at a similar level to the *Bmpr1a* cKO mice. (*F*) Expression levels of *Osx* and *Dspp* in mandibular molars were measured by qRT‐PCR. The expression levels of *Osx* and *Dspp* in the compound mutant molars were similar to those in the cKO molars. Values represent the mean ± SD. Differences were assessed by one‐way ANOVA, followed by a Tukey test, *n* = 6, *p < 0.05, **p < 0.01, ***p < 0.001, ****p < 0.0001. ns = Not significant.

To further elucidate the function of BMP‐Smad signaling in dentin formation, we generated *Bmpr1a* cKO mice also expressing *caBmpr1a* to restore only Smad signaling activity (compound mutant mice). The mandibulae from the *Bmpr1a* cKO and compound mutant mice were smaller than those from controls, but no eruption problem was observed among three groups at P21 (Fig. 4 *A*–*C*). {FIG4} The cKO and compound mutant mice displayed short roots compared with controls (Fig. 4 *A’*–*C’*). The volumetric analysis demonstrated that both crown and root dentin volume in the cKO molars were lower than that in controls, whereas no significant difference in the crown dentin volume was observed between the compound mutant and control molars (Fig. 4 *D*), suggesting that restoring BMP‐Smad signaling by *caBmpr1a* partially rescued the cKO molar phenotype. However, root dentin volume and root heights in the compound mutant molars were similar with those in the cKO molars (Fig. 4 *E*). These findings suggest that BMP‐Smad signaling in odontoblasts regulates crown dentin formation but not root dentin formation. However, we did not observe any changes in *Osx* or *Dspp* expression levels between the cKO and compound mutant molars (Fig. 4 *F*).

**Figure 5:**
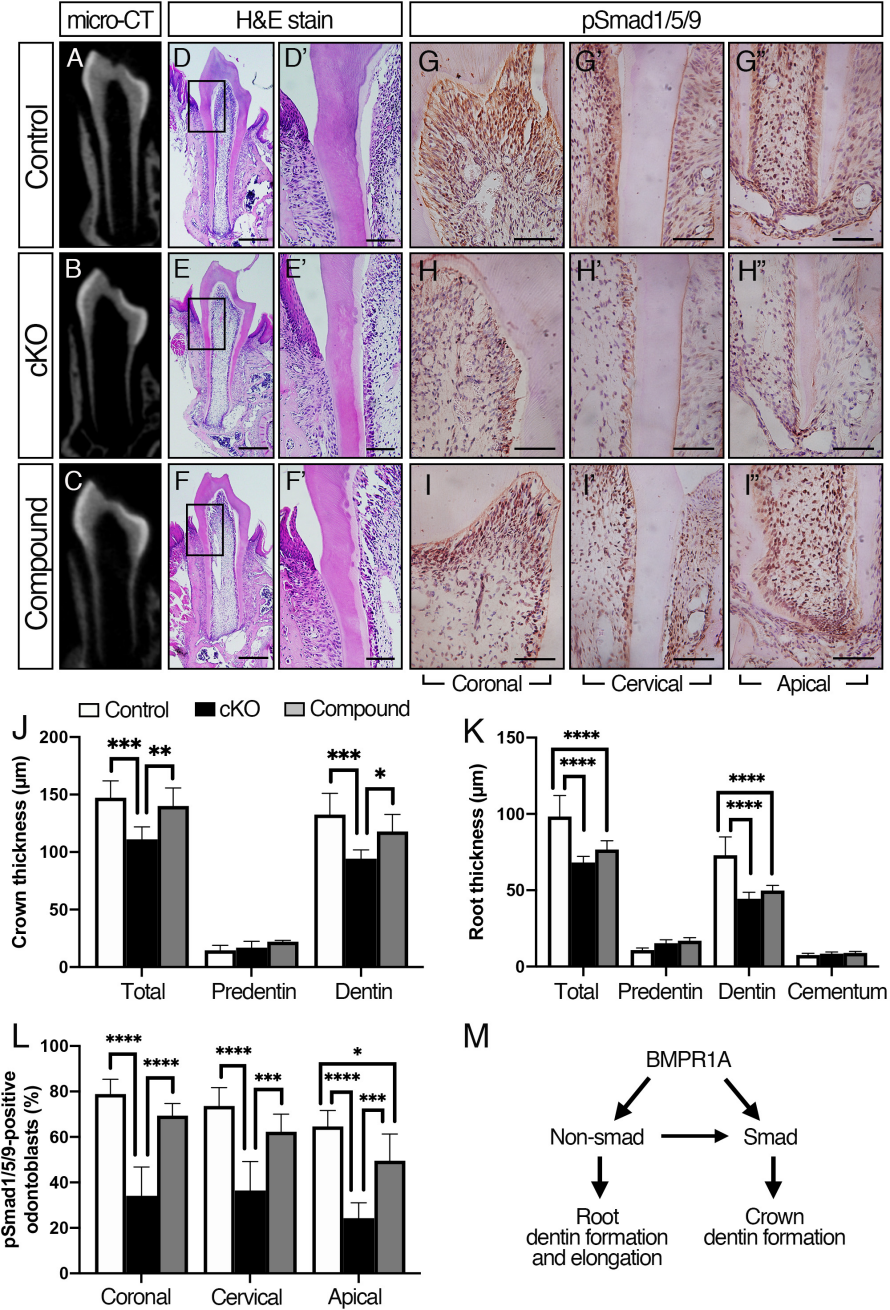
Restoring Smad1/5/9 signaling activity in the compound mutant mice rescues the function of odontoblasts in the crown. (*A*–*C*) Representative micro‐CT coronal views of the mandibular first molar at P21. (*D*–*F*) Representative coronal images of H&E staining of the mandibular first molars at P21. High magnification of cervical region of the first molar (*D’*–*F’*). (*G*–*I*) Representative images of pSmad1/5/9 staining of the mandibular first molars at P21. High magnification of coronal (*G*–*I*), cervical (*G’*–*I’*), and apical (*G”*–*I”*) regions of the first molar. (*J*, *K*) Quantification of crown thickness (*J*) and root thickness (*K*). The region was determined from the cemento‐enamel junction that extended 100 μm toward coronal direction (crown thickness) or apical direction (root thickness). Reduced crown dentin thickness in the *Bmpr1a* cKO molars was restored in the compound mutants. (*L*) Quantification of pSmad1/5/9‐positive odontoblasts in the coronal, cervical, and apical region of the first molars. The number of pSmad1/5/9‐positive odontoblasts were lower in the *Bmpr1a* cKO molars, but those in the compound mutant molars were higher than those in the cKO molars. (*M*) A schematic diagram showing how BMP signaling in odontoblasts regulates postnatal tooth dentin formation. Our findings suggest that BMP‐Smad signaling plays a role in crown dentinogenesis, whereas non‐Smad signaling may play a major role in root dentinogenesis and elongation. Our results also suggest that BMP‐non‐Smad signaling pathways may cooperatively work with BMP‐Smad signaling to regulate expression of dentinogenesis‐associated genes in odontoblasts. Values represent the mean ± SD. Differences were assessed by one‐way ANOVA, followed by a Tukey test, *n* = 6, *p < 0.05, **p < 0.01, ***p < 0.001, ****p < 0.0001. ns = Not significant. Scale bars = 500 μm (*D–F*), 100 μm (*D’–F’*), 50 μm (*H–J*, *H’–J’*, *H”–J”*).

Next, we conducted morphological analysis to compare crown and root thickness among control, *Bmpr1a* cKO and compound mutant groups (Fig. 5 *A–F*). {FIG5} The coronal view of micro‐CT images and H&E staining showed reduced dentin thickness and short roots in the cKO mice. Consistent with the volumetric analysis, decreased dentin thickness in the crown region of the cKO molars was restored in the compound mutant molars; however, dentin in the root region was thinner both in the cKO and rescued groups than that in controls (Fig. 5 *J, K*). Interestingly, compound mutant mice showed the irregular dentin‐predentin boundary in the crown region (Fig. 5 *D’–F’*). We observed reduced levels of pSmad1/5/9 in coronal, cervical, and apical regions of odontoblasts in the cKO molars compared with controls, whereas pSmad1/5/9 levels in coronal and cervical regions of odontoblasts in the compound mutant molars were similar to those in the controls (Fig. 5 *G*–*I*, *G’*–*I’, G”*–*I”, L*). The number of pSmad1/5/9‐positive odontoblasts in the apical region of compound mutant molars were significantly higher than those in the cKO molars with slightly lower levels compared with those in controls. These results suggest that restoring Smad1/5/9 signaling activity can only rescue the function of odontoblasts in the crown.

## Discussion

Our findings demonstrate that loss of BMP signaling in odontoblasts results in impaired crown and root dentin formation, and short roots associated with reduced *Osx* and *Dspp* expression. Compared with crown dentinogenesis, heterogeneous populations of odontoblasts contribute to root dentinogenesis. Restoring Smad activity in the compound mutant mice rescues reduced crown dentin formation in the *Bmpr1a* cKO mice; however, impaired root dentin formation and short roots are not rescued. Taken together, BMP‐Smad signaling plays a role in crown dentinogenesis, whereas non‐Smad signaling may play a major role in root dentinogenesis as well as root elongation (Fig. 5 *M*).

It is well known that BMP signaling plays an essential role in early tooth morphogenesis. Mice deficient for *Bmp2* in the neural crest lineage cells results in a delay in dentin and enamel deposition.^5^ Neural crest‐specific disruption of *Bmpr1a* leads to delayed tooth development at the bud/early cap stages due to impaired differentiation of dental mesenchymal cells.^23^ We and others also demonstrated that BMP signaling may play an essential role during postnatal tooth development as *Bmp2* and *Bmp4* cKO mice display reduced dentin formation.^6^, ^7^ Because BMPs are critical for early tooth development, it is difficult to address the function of BMP signaling in postnatal dentin formation with non‐inducible Cre mouse lines. In the mouse first molars, differentiated odontoblasts align to the basement membrane at P0 and start to secrete dentin matrix, and then rapid tooth growth and root elongation initiate at P6.^24^ Therefore, to understand the function of BMP signaling in crown and root dentinogenesis, we used inducible Tet‐off *Osx‐Cre* mice and induced Cre activity at the day of delivery by removing doxycycline for nursing mothers to specifically delete *Bmpr1a* in odontoblasts during postnatal development.

Recent study suggests the heterogeneity of odontoblasts between crown and root in molars.^3^ Morphologically, mature odontoblasts in the crown are elongated and have a cylindrical shape, but they are cubic in the root region. Pre‐odontoblasts located in the apical region are small ovoid cells.^25^ *Osx*‐expressing cells at the early postnatal stage differentiate into both odontoblasts and cementoblasts in the root and thus contribute to root formation.^26^ Here, we showed that crown dentin was formed by *Osx*‐expressing odontoblasts, whereas root dentin was formed by *Osx*‐expressing and *Osx*‐negative odontoblasts during primary dentinogenesis. Additionally, newly differentiated *Osx*‐negative odontoblasts were responsible for secondary dentinogenesis in the crown. Because we applied inducible Tet‐off *Osx‐Cre* and induced Cre activity at the day of birth to knockout *Bmpr1a* in odontoblasts, it would be possible that doxycycline was accumulated in the tissues, which prevented Cre recombination in the root odontoblasts. As shown in Fig. 1, however, GFP‐positive odontoblasts were observed in the crown at P5 and root at P14, suggesting successful recombination both in the crown and root odontoblasts. Additionally, primary dentin in the crown was formed by *Osx*‐expressing odontoblasts, whereas secondary dentin that is formed after root formation is complete was formed by *Osx*‐negative odontoblasts. These results suggest that differences observed in the crown and root dentin and primary and secondary dentin are not due to different recombination efficiency caused by doxycycline accumulation, but due to different populations of odontoblasts contribute to distinct dentin formation at a stage‐ and site‐specific manner. It is well known that cranial neural crest cells directly differentiate into cells in the dental papilla and odontoblasts during embryonic development. However, a recent study demonstrated that some of the dental mesenchymal cells are derived from peripheral glial cells, which are supplied by innervation during postnatal tooth development.^27^ Another group reported that dental pericytes residing in the microvasculature can differentiate into odontoblasts during tooth repair.^28^ However, those studies were conducted on the incisor, and thus their contributions to molar development are still unknown. Therefore, investigating the involvement of different populations of odontoblasts in site‐ and stage‐specific dentinogenesis in molars will deepen our understanding of a complex process of tooth organogenesis.

How BMP‐Smad and BMP‐non‐Smad signaling coordinately regulate postnatal crown and root dentin formation has not been well understood. The deletion of *Smad4* in odontoblasts, a common mediator of the TGF‐β/BMP signaling pathways, leads to delayed odontoblast differentiation and irregular dentin formation,^10^, ^11^ suggesting that BMP‐Smad signaling plays a critical role in dentinogenesis. Here, we showed that the loss of BMP signaling results in reduced crown and root dentin formation, but restoring Smad signaling activity only rescued crown dentin formation, suggesting that BMP‐Smad signaling regulates crown dentinogenesis. Previous studies show that p38 MAPK signaling is involved in BMP‐2‐induced odontoblast differentiation of human dental pulp cells.^29^, ^30^ p38 is highly expressed in odontoblasts during primary dentinogenesis, but it is downregulated during secondary dentinogenesis,^31^ suggesting that p38 signaling may be involved in primary dentinogenesis; however, its role in dentin formation in vivo by genetic ablation of p38 has not yet been reported. Besides, TGF‐β activated kinase1 (TAK1) acts as an important signal transducer for non‐Smad signaling pathways. We reported that *Tak1*‐deficient mutants display a round skull, hypoplastic maxilla and mandible, and cleft palate.^32^ Interestingly, deletion of *Tak1* aggravates the craniofacial deformities of *caBmpr1a;P0‐Cre* mutants that have craniosynostosis.^33^ Those cKO mice die during the perinatal period precluding functional assessment of TAK1 during postnatal tooth development including secondary dentin formation; therefore, addressing the function of TAK1 with inducible gene targeting systems will provide additional insights into how BMP‐non‐Smad signaling regulates postnatal dentinogenesis.

Osx, a zinc finger‐containing transcription factor identified as a BMP‐2 inducible gene, plays an essential role in bone and tooth formation.^34^ Biochemical study has identified a Smad binding element (SBE) between −552 and −839 bp in the *Osx* promoter and thus BMP‐Smad induces *Osx* expression.^35^ Dentin sialophosphoprotein (DSPP) also plays a critical role in proper dentin mineralization as *Dspp* KO mice display hypomineralized teeth and reduced dentin formation.^36^ Recent study shows that BMP‐2 activates *Dspp* transcription and that is mediated by OSX in vitro.^30^ Additionally, Smad1/5/9 directly binds to SBEs in the *Dspp* promoter from −211 to −183 bp and activates *Dspp* transcription.^37^ These findings suggest that canonical BMP‐Smad signaling pathway plays a key role in regulating *Osx* and *Dspp* transcription. Here, we showed that the loss of BMP signaling in odontoblasts decreased *Osx* and *Dspp* expression, suggesting that BMP signaling via BmpR1A regulates *Osx* and *Dspp* expression to promote dentin formation and mineralization. It is also reported that MAPK signaling activity modulates BMP‐induced *Osx* expression in osteoblast‐lineage cells.^38^, ^39^ Activation of the BMP‐Smad signaling leads to increased levels of Dlx5, which further phosphorylated by BMP‐2‐mediated p38 to regulate *Osx* expression.^38^ BMP‐mediated p38 activity also enhances transcriptional activation of Osx through its phosphorylation on Ser‐73/77.^39^ These findings suggest that Smad and non‐Smad signaling pathways coordinately regulate *Osx* expression. Interestingly, we found that reduced *Osx* and *Dspp* expression in the cKO molars remained unchanged in the compound mutant molars even though increased Smad1/5/9 signaling activity restored impaired crown dentin formation. These results suggest that BMP‐non‐Smad signaling activity may be required for BMP‐Smad‐induced *Osx* and *Dspp* expression.

Tertiary dentin is rapidly produced by odontoblasts in response to external stimulation such as cavities and wear. It contains entrapped odontoblasts and few dentinal tubules, thereby resembling bone called osteodentin. Interestingly, BMP‐2 deletion in odontoblasts results in osteodentin formation in molar roots.^6^ The deletion of AcvR1, another BMP type 1 receptor, results in osteodentin formation in the incisor but not in the molar,^40^ suggesting that loss of BMP signaling alters odontoblast phenotype in a site‐specific manner. Here, however, we did not observe osteodentin in the *Bmpr1a* cKO molars during primary dentin formation. This may be due to the different function of BMP signaling mediated by different type 1 receptors or due to the timing of inactivation of BMP signaling, as we induced Cre recombination at the day of birth in the *Bmpr1a* cKO mice but during embryogenesis in the *Acvr1* cKO mice. Further elucidating the cellular and molecular mechanisms that control stage‐ and site‐specific dentin formation would be required to understand physiopathology of dentinogenesis.

The mechanism by which BMP signaling in odontoblasts regulates root elongation remains unclear. The cells in HERS play an essential role in odontoblast differentiation and root elongation.^41^ BMPs in the dental mesenchyme may participate in the formation of the HERS because BMPs are expressed strongly before root development.^42^ NFI‐C has been shown as an important factor for root elongation as *Nfic* KO mice display no root formation.^2^, ^19^, ^20^ Here, we observed no difference in the *Nfic* expression between *Bmpr1a* cKO and control molars. Instead, we found increased Ki67‐positive cells and p‐Histone H3‐positive cells in the cKO molars, suggesting that a higher cell proliferation activity that may lead to failure of progenitor cells to differentiate into odontoblasts. Similarly, odontoblast‐specific disruption of *Osx* using *Col1‐Cre* transgenic mice display an increase in cell proliferation and a decrease in *Dspp* expression, leading to inhibition of odontoblast differentiation and short roots,^2^ suggestive of involvement of BMP‐mediated *Osx* expression in regulating root formation. Moreover, disruption of *Bmpr1a* in *Osx*‐expressing cells results in short roots, whereas mice lacking *Bmpr1a* in *Gli1*‐expresing cells display no root formation in molars,^43^ suggesting the important function of *Bmpr1a* in the dental mesenchyme for root formation. The *Gli1‐Cre* marks early odontoblast progenitor cells compared with *Osx‐Cre* but also marks some dental epithelial populations.^44^ Tooth development is severely affected in *K14‐Cre;Bmpr1a* cKO mice as they display a complete absence of incisor and molar tooth structures,^45^ suggesting a critical function of *Bmpr1a* in the dental epithelium. Further addressing whether deletion of BmpR1A signaling in the dental mesenchyme directly influences root elongation or secondarily affects HERS growth through impaired odontoblast differentiation will be needed to understand the role of *Bmpr1a* in root formation.

In summary, BMP signaling mediated by BmpR1A plays an important role in dentinogenesis during postnatal tooth development. The different populations of odontoblasts contribute to crown and root dentinogenesis in molars. BMP‐Smad signaling primarily regulates crown dentin formation, whereas BMP‐non‐Smad signaling may play a major role in root dentin formation and elongation during primary dentinogenesis.

## Disclosures

All authors state that they have no conflicts of interest.

## Supporting information


**Supplemental Table S1.** Primary Antibodies and Dilutions
**Supplemental Table S2.** Sequences for qRT‐PCR Primers

## References

[1] Zilberman U , Smith P . Sex‐ and age‐related differences in primary and secondary dentin formation. Adv Dent Res. 2001;15:42–5. 12640738 10.1177/08959374010150011101

[2] Zhang H , Jiang Y , Qin C , Liu Y , Ho SP , Feng JQ . Essential role of osterix for tooth root but not crown dentin formation. J Bone Miner Res. 2015;30(4):742–6. 25349111 10.1002/jbmr.2391 PMC4617775

[3] Bae CH , Kim TH , Chu JY , Cho ES . New population of odontoblasts responsible for tooth root formation. Gene Expr Patterns. 2013;13(5–6):197–202. 23603379 10.1016/j.gep.2013.04.001

[4] Jia S , Zhou J , Gao Y , et al. Roles of Bmp4 during tooth morphogenesis and sequential tooth formation. Development. 2013;140(2):423–32. 23250216 10.1242/dev.081927 PMC3597212

[5] Malik Z , Alexiou M , Hallgrimsson B , Economides AN , Luder HU , Graf D . Bone morphogenetic protein 2 coordinates early tooth mineralization. J Dent Res. 2018;97(7):835–43. 29489425 10.1177/0022034518758044

[6] Rakian A , Yang WC , Gluhak‐Heinrich J , et al. Bone morphogenetic protein‐2 gene controls tooth root development in coordination with formation of the periodontium. Int J Oral Sci. 2013;5(2):75–84. 23807640 10.1038/ijos.2013.41 PMC3707077

[7] Yang W , Harris MA , Cui Y , Mishina Y , Harris SE , Gluhak‐Heinrich J . Bmp2 is required for odontoblast differentiation and pulp vasculogenesis. J Dent Res. 2012;91(1):58–64. 21984706 10.1177/0022034511424409 PMC3232115

[8] Heldin CH , Moustakas A . Signaling receptors for TGF‐beta family members. Cold Spring Harb Perspect Biol. 2016;8:a022053. https://cshperspectives.cshlp.org/content/8/8/a022053.abstract?sid=72a1083d‐a375‐4e41‐8e92‐f05de1acb096 10.1101/cshperspect.a022053 PMC4968163 27481709

[9] Katagiri T , Watabe T . Bone morphogenetic proteins. Cold Spring Harb Perspect Biol. 2016;8:a021899. https://cshperspectives.cshlp.org/content/8/6/a021899.full 10.1101/cshperspect.a021899 PMC4888821 27252362

[10] Gao Y , Yang G , Weng T , et al. Disruption of Smad4 in odontoblasts causes multiple keratocystic odontogenic tumors and tooth malformation in mice. Mol Cell Biol. 2009;29(21):5941–51. 19703995 10.1128/MCB.00706-09 PMC2772727

[11] Kim TH , Bae CH , Lee JY , et al. Temporo‐spatial requirement of Smad4 in dentin formation. Biochem Biophys Res Commun. 2015;459(4):706–12. 25770424 10.1016/j.bbrc.2015.03.014

[12] Li J , Huang X , Xu X , et al. SMAD4‐mediated WNT signaling controls the fate of cranial neural crest cells during tooth morphogenesis. Development. 2011;138(10):1977–89. 21490069 10.1242/dev.061341 PMC3082303

[13] Mishina Y , Hanks MC , Miura S , Tallquist MD , Behringer RR . Generation of Bmpr/Alk3 conditional knockout mice. Genesis. 2002;32(2):69–72. 11857780 10.1002/gene.10038

[14] Rodda SJ , McMahon AP . Distinct roles for hedgehog and canonical Wnt signaling in specification, differentiation and maintenance of osteoblast progenitors. Development. 2006;133(16):3231–44. 16854976 10.1242/dev.02480

[15] Kamiya N , Ye L , Kobayashi T , et al. BMP signaling negatively regulates bone mass through sclerostin by inhibiting the canonical Wnt pathway. Development. 2008;135(22):3801–11. 18927151 10.1242/dev.025825 PMC2694443

[16] Kamiya N , Kobayashi T , Mochida Y , et al. Wnt inhibitors Dkk1 and Sost are downstream targets of BMP signaling through the type IA receptor (BMPRIA) in osteoblasts. J Bone Miner Res. 2010;25(2):200–10. 19874086 10.1359/jbmr.090806 PMC3153381

[17] Yushkevich PA , Piven J , Hazlett HC , et al. User‐guided 3D active contour segmentation of anatomical structures: significantly improved efficiency and reliability. Neuroimage. 2006;31(3):1116–28. 16545965 10.1016/j.neuroimage.2006.01.015

[18] Ono W , Sakagami N , Nishimori S , Ono N , Kronenberg HM . Parathyroid hormone receptor signalling in osterix‐expressing mesenchymal progenitors is essential for tooth root formation. Nat Commun. 2016;7:11277. 27068606 10.1038/ncomms11277 PMC4832076

[19] Lee HK , Lee DS , Park SJ , Cho KH , Bae HS , Park JC . Nuclear factor I‐C (NFIC) regulates dentin sialophosphoprotein (DSPP) and E‐cadherin via control of Kruppel‐like factor 4 (KLF4) during dentinogenesis. J Biol Chem. 2014;289(41):28225–36. 25138274 10.1074/jbc.M114.568691 PMC4192478

[20] Liu Y , Feng J , Li J , Zhao H , Ho TV , Chai Y . An Nfic‐hedgehog signaling cascade regulates tooth root development. Development. 2015;142(19):3374–82. 26293299 10.1242/dev.127068 PMC4631759

[21] Komatsu Y , Yu PB , Kamiya N , et al. Augmentation of Smad‐dependent BMP signaling in neural crest cells causes craniosynostosis in mice. J Bone Miner Res. 2013;28(6):1422–33. 23281127 10.1002/jbmr.1857 PMC3638058

[22] Hayano S , Komatsu Y , Pan H , Mishina Y . Augmented BMP signaling in the neural crest inhibits nasal cartilage morphogenesis by inducing p53‐mediated apoptosis. Development. 2015;142(7):1357–67. 25742798 10.1242/dev.118802 PMC4378250

[23] Li L , Lin M , Wang Y , Cserjesi P , Chen Z , Chen Y . BmprIa is required in mesenchymal tissue and has limited redundant function with BmprIb in tooth and palate development. Dev Biol. 2011;349(2):451–61. 21034733 10.1016/j.ydbio.2010.10.023 PMC3019275

[24] Lungova V , Radlanski RJ , Tucker AS , Renz H , Misek I , Matalova E . Tooth‐bone morphogenesis during postnatal stages of mouse first molar development. J Anat. 2011;218(6):699–716. 21418206 10.1111/j.1469-7580.2011.01367.x PMC3125904

[25] Sasaki T , Garant PR . Structure and organization of odontoblasts. Anat Rec. 1996;245(2):235–49. 8769666 10.1002/(SICI)1097-0185(199606)245:2<235::AID-AR10>3.0.CO;2-Q

[26] Takahashi A , Ono N , Ono W . The fate of Osterix‐expressing mesenchymal cells in dental root formation and maintenance. Orthod Craniofac Res. 2017;20(Suppl 1):39–43. 28643909 10.1111/ocr.12167 PMC5787343

[27] Kaukua N , Shahidi MK , Konstantinidou C , et al. Glial origin of mesenchymal stem cells in a tooth model system. Nature. 2014;513(7519):551–4. 25079316 10.1038/nature13536

[28] Feng J , Mantesso A , De Bari C , Nishiyama A , Sharpe PT . Dual origin of mesenchymal stem cells contributing to organ growth and repair. Proc Natl Acad Sci U S A. 2011;108(16):6503–8. 21464310 10.1073/pnas.1015449108 PMC3081015

[29] Qin W , Liu P , Zhang R , et al. JNK MAPK is involved in BMP‐2‐induced odontoblastic differentiation of human dental pulp cells. Connect Tissue Res. 2014;55(3):217–24. 24409810 10.3109/03008207.2014.882331

[30] Yang J , Ye L , Hui TQ , et al. Bone morphogenetic protein 2‐induced human dental pulp cell differentiation involves p38 mitogen‐activated protein kinase‐activated canonical WNT pathway. Int J Oral Sci. 2015;7(2):95–102. 26047580 10.1038/ijos.2015.7 PMC4817555

[31] Simon S , Smith AJ , Lumley PJ , et al. Molecular characterization of young and mature odontoblasts. Bone. 2009;45(4):693–703. 19555781 10.1016/j.bone.2009.06.018

[32] Yumoto K , Thomas PS , Lane J , et al. TGF‐beta‐activated kinase 1 (Tak1) mediates agonist‐induced Smad activation and linker region phosphorylation in embryonic craniofacial neural crest‐derived cells. J Biol Chem. 2013;288(19):13467–80. 23546880 10.1074/jbc.M112.431775 PMC3650384

[33] Liu X , Hayano S , Pan H , et al. Compound mutations in Bmpr1a and Tak1 synergize facial deformities via increased cell death. Genesis. 2018;56(3):e23093. 29411501 10.1002/dvg.23093 PMC5854540

[34] Nakashima K , Zhou X , Kunkel G , et al. The novel zinc finger‐containing transcription factor osterix is required for osteoblast differentiation and bone formation. Cell. 2002;108(1):17–29. 11792318 10.1016/s0092-8674(01)00622-5

[35] Mandal CC , Drissi H , Choudhury GG , Ghosh‐Choudhury N . Integration of phosphatidylinositol 3‐kinase, Akt kinase, and Smad signaling pathway in BMP‐2‐induced osterix expression. Calcif Tissue Int. 2010;87(6):533–40. 20872216 10.1007/s00223-010-9419-3 PMC3055166

[36] Yamakoshi Y , Kinoshita S , Izuhara L , Karakida T , Fukae M , Oida S . DPP and DSP are necessary for maintaining TGF‐beta1 activity in dentin. J Dent Res. 2014;93(7):671–7. 24799420 10.1177/0022034514534690 PMC4107551

[37] Wan C , Yuan G , Luo D , et al. The dentin sialoprotein (DSP) domain regulates dental mesenchymal cell differentiation through a novel surface receptor. Sci Rep. 2016;6:29666. 27430624 10.1038/srep29666 PMC4949421

[38] Ulsamer A , Ortuno MJ , Ruiz S , et al. BMP‐2 induces Osterix expression through up‐regulation of Dlx5 and its phosphorylation by p38. J Biol Chem. 2008;283(7):3816–26. 18056716 10.1074/jbc.M704724200

[39] Ortuno MJ , Ruiz‐Gaspa S , Rodriguez‐Carballo E , et al. p38 regulates expression of osteoblast‐specific genes by phosphorylation of osterix. J Biol Chem. 2010;285(42):31985–94. 20682789 10.1074/jbc.M110.123612 PMC2952199

[40] Zhang X , Shi C , Zhao H , et al. Distinctive role of ACVR1 in dentin formation: requirement for dentin thickness in molars and prevention of osteodentin formation in incisors of mice. J Mol Histol. 2019;50(1):43–61. 30519900 10.1007/s10735-018-9806-z

[41] Huang X , Bringas P Jr , Slavkin HC , Chai Y . Fate of HERS during tooth root development. Dev Biol. 2009;334(1):22–30. 19576204 10.1016/j.ydbio.2009.06.034 PMC2744848

[42] Yamashiro T , Tummers M , Thesleff I . Expression of bone morphogenetic proteins and Msx genes during root formation. J Dent Res. 2003;82(3):172–6. 12598544 10.1177/154405910308200305

[43] Feng J , Jing J , Li J , et al. BMP signaling orchestrates a transcriptional network to control the fate of mesenchymal stem cells in mice. Development. 2017;144(14):2560–9. 28576771 10.1242/dev.150136 PMC5536932

[44] Seidel K , Ahn CP , Lyons D , et al. Hedgehog signaling regulates the generation of ameloblast progenitors in the continuously growing mouse incisor. Development. 2010;137(22):3753–61. 20978073 10.1242/dev.056358 PMC3049275

[45] Andl T , Ahn K , Kairo A , et al. Epithelial Bmpr1a regulates differentiation and proliferation in postnatal hair follicles and is essential for tooth development. Development. 2004;131(10):2257–68. 15102710 10.1242/dev.01125

