# Supplementary material for: BMP‐Smad Signaling Regulates Postnatal Crown Dentinogenesis in Mouse Molar
Source: JBMR Plus. 2019 Nov 14;4(2):e10249. doi: 10.1002/jbm4.10249 (PMC7017888; doi:10.1002/jbm4.10249)
Supplement: Supplementary file 1 — Supplemental Table S1. Primary Antibodies and Dilutions Supplemental Table S2. Sequences for qRT‐PCR Primers [file JBM4-4-e10249-s001.docx]

**BMP-Smad Signaling Regulates Postnatal Crown Dentinogenesis in Mouse Molar**

Maiko Omi^1*^, Anshul K. Kulkarni^1^, Anagha Raichur^1^, Mason Fox^1^, Amber Uptergrove^1^, Honghao Zhang^1^, and Yuji Mishina^1*^

**Supplemental Tables**

**Table S1. Primary antibodies and dilutions**

rabbit anti-phospho-Smad1/5/9 (1:200 for IHC, 1:1000 for WB, Cell Signaling, 13820)

rabbit anti-phospho-p44/42 MAPK (ERK) (1:1000, Cell Signaling, 4376),

rabbit anti-p44/42 MAPK (ERK) (1:1000, Cell Signaling, 4695)

rabbit anti-phospho-p38 MAPK (1:1000, Cell Signaling, 4631)

rabbit anti-p38 MAPK (1:1000, Cell Signaling, 9212)

rabbit anti-GAPDH (1:2000, Cell Signaling, 2118)

**Table S2. Sequences for qRT-PCR primers**

*Sp7/Osterix* F 5’- GGGCGTTCTACCTGCGACTG -3’

R 5’- ATCGGGGCGGCTGATTG -3’

*Dspp* F 5’- AGTGTGGAAAGTGTGGCGTT -3’

R 5’- CTGTTGCTAGTGGTGCTGTT -3’

*Nfic* F 5’- GACCTGTACCTGGCCTACTTTG -3’

R 5’- CACACCTGACGTGACAAAGCTC -3’

*Nestin* F 5’- AATGGGAGGATGGAGAATGGAC -3’

R 5’- TAGACAGGCAGGGCTAGCAAG -3’

*Bsp* F 5’- CAGGGAGGCAGTGACTCTTC -3’

R 5’- AGTGTGGAAAGTGTGGCGTT -3’

*Dmp1* F 5’- CATTCTCCTTGTGTTCCTTTGGG -3’

R 5’- TGTGGTCACTATTTGCCTGTG -3’

*Spp1/Osteopontin* (*Opn*) F 5’- TCTCCTTGCGCCACAGAATG -3’

R 5’- TCCTTAGACTCACCGCTCTT -3’

*Bglap2/Ostecalcin* (*Oc*) F 5’- AGACAAGTCCCACACAGCA -3’

R 5’- CTGCTTGGACATGAAGGC -3’

*Col1a1* F 5'- TCTCCACTCTTCTAGGTTCCT -3'

R 5'- TTGGGTCATTTCCACATGC -3'

*Gapdh* F 5’- CGTCCCGTAGACAAAATGGT -3’

R 5’- TTGATGGCAACAATCTCCAC -3’
